# Prognostic factors associated with visual field recovery in non-arteritic anterior ischemic optic neuropathy

**DOI:** 10.1371/journal.pone.0351219

**Published:** 2026-06-12

**Authors:** Heejin Yoon, Su-Jin Kim, Seung Min Lee, Sangwoo Moon, JungHyo Ahn, Jae Hwan Choi, Ji Eun Lee

**Affiliations:** 1 Department of Ophthalmology, Pusan National University Yangsan Hospital, Pusan National University School of Medicine, Yangsan, South Korea; 2 Research Institute for Convergence of Biomedical Science and Technology, Pusan National University Yangsan Hospital, Yangsan, South Korea; 3 Department of Neurology, Pusan National University Yangsan Hospital, Pusan National University School of Medicine, Yangsan, South Korea; Daegu Veterans Health Service Medical Center, KOREA, REPUBLIC OF

## Abstract

This study aimed to identify baseline prognostic factors associated with visual field (VF) recovery in patients with non-arteritic anterior ischemic optic neuropathy (NAION). This retrospective study included 38 patients (41 eyes) diagnosed with NAION. VF recovery was defined as an improvement of ≥3 dB in mean deviation (MD) at 3 months. Baseline demographic characteristics, systemic vascular risk factors, visual function tests, and retinal nerve fiber layer (RNFL) thickness measured by optical coherence tomography (OCT) were analyzed. Eleven eyes (26.8%) were classified as the recovery group. Compared with the non-recovery group, the recovery group had significantly better baseline best-corrected visual acuity (BCVA) (P = 0.034) and lower glycated hemoglobin levels (P = 0.033). Baseline VF impairment was significantly less severe in the recovery group, with higher MD (P = 0.049) and visual field index (VFI) values (P = 0.014). No significant association was observed between topical eye drop treatment, including brimonidine and netarsudil, and visual field recovery (p = 0.723). Baseline BCVA strongly correlated with BCVA at 1, 3, and 6 months (all P < 0.001). Multivariate analysis confirmed that better baseline MD (P = 0.041) and higher VFI (P = 0.036) were independently associated with VF recovery. However, RNFL thickness measured at initial visit by OCT did not reliably predict recovery. These findings highlight that baseline functional status remain as the most important predictor of visual field recovery in NAION.

## Introduction

Non-arteritic anterior ischemic optic neuropathy (NAION) is the most common acute optic neuropathy in middle-aged and older adults and a major cause of sudden, painless visual loss and visual field (VF) defects [1,2]. NAION is also the most common cause of acute vision loss related to optic nerve disease. Although the incidence of NAION is relatively high compared with that of other optic neuropathies, the visual prognosis remains unpredictable, and no treatment has been definitively shown to alter its natural course [3–5].

Clinically, NAION is generally associated with an unfavorable visual prognosis, and many patients experience persistent VF defects even after visual acuity stabilizes. Nevertheless, a subset of patients demonstrates partial VF recovery during follow-up. The factors that distinguish these patients from those with persistent visual dysfunction remain incompletely understood. Because no treatment with proven efficacy has been established, understanding the natural history of visual recovery is of paramount importance in NAION [5–7]. From the perspective of both patients and ophthalmologists, knowledge of the expected course of visual function is essential for counseling and management.

In this study, therefore, we aimed to systematically analyze prognostic factors associated with VF recovery in patients with NAION. Recovery of mean deviation (MD) on VF testing was used as the primary outcome for prognostic analysis.

## Methods

This retrospective study reviewed the medical records of patients diagnosed with NAION at Pusan National University Yangsan Hospital between January 2017 and December 2024. The study was conducted in accordance with the Declaration of Helsinki and was approved by the Institutional Review Board of Pusan National University Yangsan Hospital (IRB number: 55-2026-062). Due to the study’s retrospective nature, the requirement for informed consent was waived by the Institutional Review Board. The data were accessed for research purposes from March 18 to March 20, 2026.

Patients were included if they had a clinical diagnosis of NAION confirmed by ophthalmologic examination and completed at least 3 months of follow-up. NAION was diagnosed based on established clinical criteria, including acute painless unilateral visual loss, optic disc edema at presentation, optic disc–related visual field defects, and the absence of any systemic or ocular disease that could account for the findings [5,6,8]. Eligibility required comprehensive baseline ophthalmic assessments, including best-corrected visual acuity (BCVA), automated VF testing using a VF analyzer (Humphrey Field Analyzer; Carl Zeiss-Meditec, Dublin, CA, USA), and optical coherence tomography (OCT) performed with the Cirrus HD OCT® (Carl Zeiss-Meditec, Oberkochen, Germany).

Patients were excluded if they had any of the following conditions: (1) arteritic anterior ischemic optic neuropathy, including giant cell arteritis; (2) a history of glaucoma or other optic nerve diseases that could affect VF interpretation; (3) retinal diseases associated with VF defects; (4) neurological disorders involving the visual pathway including cerebral ischemia or infarction confirmed on MRI; or (5) incomplete clinical or imaging data.

Patients who demonstrated an improvement of ≥3 dB in MD at 3 months compared with the initial visit were classified into the recovery group. All statistical analyses were performed using SPSS for Windows (version 28.0; IBM Corp., Armonk, NY, USA). Baseline demographic and clinical variables, including age, sex, visual acuity, and VF parameters, were summarized using descriptive statistics. Comparisons between the recovery and non-recovery groups were performed using independent t-tests and chi-square tests. Correlations among BCVA, VF indices, and retinal nerve fiber layer (RNFL) thickness were assessed using Pearson correlation analysis. RNFL thickness was measured at initial visit using the optic disc cube 200 × 200 scan protocol with the built-in software of the Cirrus HD-OCT (Carl Zeiss Meditec), and average and four-quadrant sectoral values were analyzed. Univariate and multivariate analyses were performed to identify prognostic factors associated with VF recovery. A two-sided P-value < 0.05 was considered statistically significant.

## Results

A total of 38 patients (38 eyes) were included in this study. Among them, three patients had bilateral involvement, he first affected eye was selected as the representative eye. Among the 38 patients, 25 were male and 13 were female. The mean age was 63.39 ± 10.82 years (36–84 years). The mean baseline BCVA was 0.55 ± 0.87 logMAR (−0.2 to 3.0), and the mean symptom duration prior to the first visit was 3.29 ± 3.35 weeks (0.5 to 16.0 weeks). Hypertension was present in 20 patients (52.6%), diabetes mellitus (DM) in 7 (18.4%), dyslipidemia in 11 (28.9%), and chronic kidney disease (CKD) in 2 (5.3%). Regarding treatment, 11 patients (28.9%) received Rhopressa (netarsudil 0.02%; Aerie Pharmaceuticals, Bedminster, NJ, USA) and Alphagan P (brimonidine 0.15%; Allergan Inc., USA) eye drops, 14 patients (36.8%) used Alphagan P alone, and 13 patients (34.2%) did not receive any topical neuroprotective eye drops (Table 1).

**Table 1:** Patient baseline characteristics.

Variable	Value
Eyes/ Patients	38/ 38
Sex (male/ female)	25/ 13
Age (years)	63.39 ± 10.82 (36.0–84.0)
Body weight (kg)	64.59 ± 10.20 (45.0–85.0)
BCVA (logMAR) at first visit	0.55 ± 0.87 (−0.2–3.0)
Duration of symptoms (weeks)	3.29 ± 3.35 (0.5–16.0)
Systemic BP	136.35 ± 16.74 (110.0–174.0)
Diastolic BP	84.10 ± 9.75 (70.0–104.0)
HbA1c	5.95 ± 0.86 (5.2–9.1)
LDL	93.04 ± 38.36 (20.0–186.0)
HDL	55.74 ± 16.57 (31.0–93.0)
TC	178.39 ± 41.78 (81.0–261.0)
TG	143.21 ± 73.83 (65.0–287.0)
Smoking	6 (15.8%)
Treatment (RA/A/None)	11 (28.9%)/ 14 (36.8%)/ 13 (34.2%)
Visual field test at the first visit	
MD (dB)	−15.48 ± 9.63 (−30.7– − 0.6)
PSD (dB)	9.07 ± 3.55 (2.4–17.3)
VFI (%)	56.84 ± 31.63 (0.0–99.0)
RNFL thickness (μm) at the first visit	
Average	175.63 ± 76.73 (70.0–359.0)
Superior quadrant	209.58 ± 93.82 (69.0–433.0)
Temporal quadrant	113.71 ± 63.78 (37.0–290.0)
Inferior quadrant	235.50 ± 115.27 (92.0–494.0)
Nasal quadrant	143.39 ± 78.03 (53.0–328.0)
Accompanying systemic disease, n(%)	
Hypertension, n (%)	20 (52.6%)
Diabetes Mellitus, n (%)	7 (18.4%)
Dyslipidemia, n (%)	11 (28.9%)
CKD, n (%)	2 (5.3%)

Values are presented as mean ± standard deviation (min-max), number, or number (%) unless otherwise indicated.

BCVA = best-corrected visual acuity; logMAR = logarithm of the minimum angle of resolution; BP = Blood Pressure; HbA1c = glycated hemoglobin; LDL = low-density lipoprotein; HDL = high-density lipoprotein; TC = Total Cholesterol; TG = Triglycerides; RA = Rhopressa + Alphagan P group; A = Alphagan P only group; MD = mean deviation; PSD = pattern standard deviation; VFI = visual field index; RNFL = retinal nerve fiber layer; CKD = Chronic Kidney Disease.

At baseline, the MD on VF testing was −15.48 ± 9.63 dB (−30.7 to −0.6 dB), the PSD was 9.07 ± 3.55 dB (2.4 to 17.3 dB), and the VFI was 56.84 ± 31.63% (0–99%). The mean RNFL average thickness was 175.63 ± 76.73 μm (70–359 μm), with quadrant measurements of 209.58 ± 93.82 μm (superior; 69–433 μm), 113.71 ± 63.78 μm (temporal; 37–290 μm), 235.50 ± 115.27 μm (inferior; 92–494 μm), and 143.39 ± 78.03 μm (nasal; 53–328 μm).

Ten eyes (26.3%) were classified as the recovery group, and 28 eyes (73.7%) were assigned to the non-recovery group. Baseline BCVA was significantly better in the recovery group than in the non-recovery group (0.21 ± 0.39 vs. 0.68 ± 0.96, P = 0.041). However, no significant differences were observed between the two groups in terms of sex distribution, age, body weight, duration of symptoms, blood pressure, lipid profiles, smoking status, or treatment modality. The location of disc edema also did not differ significantly between the recovery and non-recovery groups (P = 0.227). Similarly, the prevalence of systemic comorbidities, including hypertension, DM, dyslipidemia, and CKD, was comparable between the two groups.

VF parameters indicated less severe impairment in the recovery group, with a higher MD (−11.54 ± 5.93 vs −16.93 ± 10.39 dB, P = 0.059) and higher VFI (72.30 ± 17.58% vs. 51.11 ± 33.94%, P = 0.019). PSD did not differ significantly between the groups. RNFL thickness also showed no significant differences between the recovery and non-recovery groups (Table 2).

**Table 2:** Comparison between the recovery and non-recovery groups.

Variable	Recovery (n = 10)	Non-recovery (n = 28)	*p*-value
Eyes	10	28	
Sex (male/ female)^†^	7/3	18/10	1.000
Age (years)	65.10 ± 12.44 (36.0–80.0)	62.79 ± 10.37 (40.0–84.0)	0.569
Body weight (kg)	66.52 ± 10.67 (52.0–85.0)	63.81 ± 10.19 (45.0–80.0)	0.536
BCVA (logMAR) at first visit	0.21 ± 0.39 (−0.2–1.0)	0.68 ± 0.96 (−0.2–3.0)	**0.041**
Duration of symptoms (weeks)	3.50 ± 2.68 (1.0–8.0)	3.21 ± 3.60 (0.5–16.0)	0.820
Systemic BP	127.14 ± 19.56 (110.0–166.0)	139.04 ± 15.25 (110.0–174.0)	0.099
Diastolic BP	77.86 ± 8.45 (70.0–90.0)	85.92 ± 9.49 (70.0–104.0)	0.053
HbA1c	5.67 ± 0.73 (5.2–7.3)	6.04 ± 0.89 (5.3–9.1)	0.331
LDL	74.12 ± 26.62 (39.0–111.0)	100.60 ± 40.24 (20.0–186.0)	0.100
HDL	54.29 ± 12.50 (40.0–69.0)	56.25 ± 18.04 (31.0–93.0)	0.793
TC	158.25 ± 28.93 (115.0–209.0)	186.45 ± 43.96 (81.0–261.0)	0.108
TG	139.62 ± 85.41 (65.0–285.0)	144.65 ± 71.07 (67.0–287.0)	0.874
Smoking	1 (10.0%)	5 (17.9%)	0.559
Treatment (RA/A/None)	3/3/4	8/11/9	0.856
Visual field test at the first visit			
MD (dB)	−11.54 ± 5.93 (−25.9–-6.7)	−16.93 ± 10.39 (−30.7– − 0.6)	0.059
PSD (dB)	8.99 ± 3.05 (3.7–13.0)	9.11 ± 3.77 (2.4–17.3)	0.929
VFI (%)	72.30 ± 17.58 (30.0–90.0)	51.11 ± 33.94 (0.0–99.0)	**0.019**
Type of disc edema			
Diffuse/Superior/Inferior/others	1/2/4/3	10/1/9/8	0.227
RNFL thickness (μm) at the first visit			
Average	158.40 ± 79.09 (72.0–349.0)	181.79 ± 76.37 (70.0–359.0)	0.416
Superior quadrant	194.90 ± 104.64 (86.0–433.0)	214.82 ± 91.12 (69.0–373.0)	0.571
Temporal quadrant	96.00 ± 32.79 (50.0–165.0)	120.04 ± 71.12 (37.0–290.0)	0.166
Inferior quadrant	207.00 ± 118.09 (92.0–494.0)	245.68 ± 114.68 (103.0–490.0)	0.370
Nasal quadrant	136.10 ± 79.05 (61.0–304.0)	146.00 ± 78.95 (53.0–328.0)	0.736
Accompanying systemic disease, n(%)			
Hypertension, n (%)	4 (40.0%)	16 (57.1%)	0.351
Diabetes Mellitus, n (%)	1 (10.0%)	6 (21.4%)	0.424
Dyslipidemia, n (%)	4 (40.0%)	7 (25.0%)	0.369
CKD, n (%)	0 (0.0%)	2 (7.1%)	1

Values are presented as mean ± standard deviation (min-max), number, or number (%) unless otherwise indicated.

BCVA = best-corrected visual acuity; logMAR = logarithm of the minimum angle of resolution; BP = Blood Pressure; HbA1c = glycated hemoglobin; LDL = low-density lipoprotein; HDL = high-density lipoprotein; TC = Total Cholesterol; TG = Triglycerides; RA = Rhopressa + Alphagan P group; A = Alphagan P only group; MD = mean deviation; PSD = pattern standard deviation; VFI = visual field index; RNFL = retinal nerve fiber layer; CKD = Chronic Kidney Disease.

Pearson correlation analyses were performed to evaluate the relationships between baseline parameters and BCVA at 1, 3, and 6 months. Baseline BCVA demonstrated strong positive correlations with BCVA at 1, 3, and 6 months (r = 0.929, 0.901, and 0.756, respectively; all P < 0.001). Baseline MD and VFI were significantly correlated with BCVA at 3 months (MD: r = −0.458, P = 0.004; VFI: r = −0.545, P < 0.001), whereas PSD showed no significant correlations. Baseline RNFL thickness was not significantly correlated with follow-up BCVA at any time point (Table 3).

**Table 3:** Relationship between baseline BCVA, visual field, RNFL thickness with follow-up BCVA.

Variable	1m BCVA		3m BCVA		6m BCVA	
	R	*p*-value^*^	R	*p*-value^*^	R	*p*-value^*^
BCVA at baseline	0.929	**<0.001**	0.901	**<0.001**	0.756	**<**0.001
Visual field test at baseline						
MD (dB)	−0.380	0.108	−0.458	**0.004**	−0.293	0.210
PSD (dB)	−0.377	0.112	−0.177	0.294	−0.012	0.959
VFI (%)	−0.435	0.063	−0.545	**<0.001**	−0.351	0.129
RNFL thickness (μm) at baseline						
Average	0.075	0.753	0.036	0.828	0.262	0.265
Superior quadrant	−0.212	0.369	−0.159	0.341	0.176	0.458
Temporal quadrant	0.221	0.349	0.097	0.562	0.434	0.056
Inferior quadrant	0.322	0.166	0.182	0.273	0.281	0.230
Nasal quadrant	−0.059	0.804	−0.012	0.942	0.058	0.809

BCVA = best-corrected visual acuity; MD = mean deviation; PSD = pattern standard deviation; VFI = visual field index; RNFL = retinal nerve fiber layer.

Univariate logistic regression analysis identified no individual variable reached statistical significance for predicting VF recovery (all P > 0.05). However, in the multivariate logistic regression model including baseline BCVA, LDL, MD, and VFI, higher baseline MD (odds ratio [OR], 0.28; 95% confidence interval [CI], 0.09–0.92; P = 0.036), higher baseline VFI (OR, 1.56; 95% CI, 1.05–2.32; P = 0.028), and lower baseline LDL (OR, 0.94; 95% CI, 0.89–1.00; P = 0.050) were independently associated with VF recovery. Baseline BCVA was not independently associated with visual recovery in the multivariate model (Table 4).Other demographic factors, systemic comorbidities, treatment modalities, and RNFL thickness were not independently associated with visual recovery (Table 4).

**Table 4:** Predictive factors for visual field recovery.

Variable	Univariate analysis			Multivariate analysis		
	OR	95% CI	*p*-value^*^	OR	95% CI	*p*-value^*^
Age (years)	1.02	0.95–1.10	0.558			
Sex (male)	1.30	0.27–6.16	0.744			
Body weight (kg)	1.03	0.94–1.12	0.521			
BCVA (logMAR) at first visit	0.39	0.10–1.53	0.177	4.633	0.35–60.84	0.243
Duration of symptoms (weeks)	1.03	0.83–1.27	0.815			
Systemic BP	0.95	0.89–1.01	0.108			
Diastolic BP	0.90	0.80–1.01	0.070			
HbA1c	0.45	0.09–2.28	0.337			
LDL	0.98	0.95–1.01	0.111	0.942	0.89-1.00	0.050
HDL	0.99	0.94–1.05	0.784			
TC	0.98	0.96–1.00	0.119			
TG	1.00	0.99–1.01	0.868			
Smoking	0.60	0.06–6.05	0.665			
Treatment (RA/A/None)			0.856			
Visual field test						
MD (dB)	1.07	0.98–1.16	0.137	0.28	0.09-0.92	**0.036**
PSD (dB)	0.99	0.80–1.22	0.926			
VFI (%)	1.03	1.00–1.06	0.081	1.56	1.05–2.32	**0.028**
Type of disc edema						
Superior vs. Diffuse						
Inferior vs. Diffuse						
Others vs. Diffuse						
RNFL thickness (μm)	1.00	0.99–1.01	0.407			
Average	1.00	0.99–1.01	0.561			
Superior quadrant	0.99	0.98–1.01	0.314			
Temporal quadrant	1.00	0.99–1.00	0.364			
Inferior quadrant	1.00	0.99–1.01	0.728			
Nasal quadrant						
Accompanying systemic disease	0.50	0.11–2.17	0.355			
Hypertension	0.41	0.04–3.88	0.435			
Diabetes Mellitus	2.00	0.43–9.21	0.374			
Dyslipidemia	~0	0–∞	1.000			
CKD						

Values are presented as mean ± standard deviation, number, or number (%) unless otherwise indicated.

BCVA = best-corrected visual acuity; logMAR = logarithm of the minimum angle of resolution; BP = Blood Pressure; HbA1c = glycated hemoglobin; LDL = low-density lipoprotein; HDL = high-density lipoprotein; TC = Total Cholesterol; TG = Triglycerides; RA = Rhopressa + Alphagan P group; A = Alphagan P only group; MD = mean deviation; PSD = pattern standard deviation; VFI = visual field index; RNFL = retinal nerve fiber layer; CKD = Chronic Kidney Disease; OR = odds ratio; CI = confidence interval.

## Discussion

The precise pathophysiology of NAION remains incompletely understood, particularly regarding the mechanisms by which ischemia develops at the optic nerve head. Multiple hypotheses have been proposed, including focal arterial insufficiency, systemic hypoperfusion, vasospasm, and dysregulation of optic nerve head microcirculation; however, direct evidence supporting any single mechanism remains limited [2–4,9]. Recent review studies increasingly characterize NAION as a multifactorial disorder rather than the consequence of a single pathogenic process, emphasizing a pathophysiological model involving structural crowding of the optic nerve head and a self-perpetuating cycle of ischemia and edema [4].

Several studies have investigated prognostic factors for visual recovery in NAION. In a large natural history study, Hayreh and Zimmerman reported that patients with milder visual acuity loss and VF defects at diagnosis were more likely to show improvement, with most recovery occurring within the first 6 months after onset [5]. Similarly, the Ischemic Optic Neuropathy Decompression Trial emphasized that the severity of initial VF loss was a key determinant of final visual outcomes, regardless of treatment [10]. Consistent with these findings, patients in the recovery group in the present study had significantly higher baseline MD and VFI values, indicating less severe VF impairment at diagnosis. Moreover, these parameters remained independently associated with VF recovery after adjustment for other variables in multivariate analysis. These results support the concept that the extent of initial functional damage reflects a “functional reserve” that influences the potential for subsequent recovery in NAION [5,10].

NAION is closely associated with systemic vascular risk factors, including hypertension, DM, dyslipidemia, and smoking, which are thought to contribute to microcirculatory dysfunction and impaired autoregulation at the optic nerve head [11–18]. However, the impact of these factors on visual recovery after disease onset has not been consistently demonstrated. In the present study, the prevalence of systemic comorbidities—including hypertension, DM, dyslipidemia, and CKD—did not differ significantly between the recovery and non-recovery groups. These findings suggest that systemic vascular risk factors are important in the pathogenesis of NAION but may have limited value in predicting visual recovery after disease onset. Although LDL levels were lower in the recovery group in univariate analysis, LDL was not an independent prognostic factor in multivariate analysis. Similarly, LDL levels were not significantly associated with long-term visual recovery in a previous study of NAION patients [19]. This may indicate that dyslipidemia influences the microvascular environment; however, the limited sample size and potential confounding factors preclude definitive conclusions.

Age has also been considered a potential prognostic factor in various optic neuropathies. Preechawat et al. reported favorable visual outcomes in NAION patients younger than 50 years [20]. Similarly, Behbehani et al. found that patients younger than 50 years were more likely to achieve better final visual acuity compared with those older than 50 years [18]. In contrast, Sun et al. did not find a significant difference in visual outcomes between patients younger and older than 55 years [21]. In the present study, patient age at diagnosis was not significantly associated with VF recovery in either univariate or multivariate analyses. Further large-scale studies are needed to determine whether age independently influences visual acuity or VF recovery in NAION, or whether, once NAION occurs, the extent of initial functional impairment plays a more critical role in determining recovery potential than chronological age alone.

Previous studies have suggested that the location of disc swelling may influence VF outcomes, as different sectors correspond to distinct retinal nerve fiber bundle distributions and VF regions [22]. In our study, we analyzed whether the predominant location of disc edema affected VF recovery; however, no significant association was identified. This discrepancy from some previous reports may reflect differences in sample size, variability in the timing of fundus evaluation during the acute phase, or the subjective nature of sectoral disc edema classification. Our findings suggest that although the location of disc involvement may influence the pattern of VF defects, it may not independently determine the likelihood of overall VF recovery.

OCT-based analysis of the RNFL is widely used to evaluate structural changes in NAION. In the acute phase, RNFL swelling occurs due to impaired axoplasmic flow, followed by irreversible RNFL thinning secondary to axonal loss [23]. Previous studies have reported inconsistent findings regarding the ability of baseline RNFL thickness to predict functional recovery [21,24]. In the present study, RNFL thickness at diagnosis was not associated with VF recovery. This finding may be explained by the fact that acute-phase RNFL measurements reflect a combination of edema and axonal loss, making it difficult to accurately predict functional prognosis based on structural parameters alone.

Topical eye drop treatment such as Rhopressa(Netarsudil) and Alphagan P(Brimonidine) was not significantly associated with visual field recovery in our study. Brimonidine, an α2-adrenergic agonist, has been suggested to exert neuroprotective effects independent of intraocular pressure (IOP) lowering, potentially through modulation of retinal ganglion cell survival and reduction of excitotoxic damage [25,26]. Similarly, netarsudil, a Rho kinase inhibitor, has been reported to improve optic nerve head perfusion and may influence microvascular dynamics, which are relevant to the ischemic pathophysiology of NAION [27]. Given that NAION is characterized by impaired microcirculation and a compartment-like effect at the optic nerve head, pharmacologic strategies targeting vascular regulation and neuronal survival remain theoretically appealing. However, in the present study, no significant difference in visual outcomes was observed according to treatment groups, which may be attributable to the limited sample size, heterogeneity in treatment initiation timing, and the retrospective nature of the study. Therefore, while our findings do not support a definitive functional benefit of these agents, they do not exclude a potential neuroprotective effect. Prospective, controlled studies with standardized treatment protocols are required to clarify whether such therapies can meaningfully preserve visual function in patients with NAION.

This study has several limitations. First, its retrospective design introduces the potential for selection and information bias. Additionally, variability in the timing of diagnosis and follow-up examinations among patients may have limited the precise characterization of changes in visual function over time. Thus, although patients with known neurological disorders involving the visual pathway were excluded, cerebral ischemic changes, including prior stroke not directly involving the visual pathway or subclinical brain ischemia, were not systematically evaluated. Thus, the potential influence of such factors on visual field recovery could not be assessed in this retrospective study. Second, the relatively small sample size may have limited statistical power, and variables that were significant in univariate analysis may not have been confirmed as independent prognostic factors in multivariate analysis. Further investigations in larger cohorts are needed to clarify the associations between systemic factors, such as HbA1c and lipid profiles, and VF recovery. Additionally, sleep apnea has been recognized as a clinically important risk factor for NAION [28]; however, data on sleep apnea diagnosis were not systematically available in the present cohort due to its retrospective nature. Future prospective studies incorporating systematic sleep apnea screening would help clarify its potential influence on visual recovery in NAION. Third, treatment assignment was not randomized and was based on the clinician’s judgment. Although no treatment has been definitively proven to be effective for NAION, caution is warranted when interpreting the effects of neuroprotective therapies. Additionally, the duration of topical treatment was not uniformly recorded across all patients [29].

Additionally, ganglion cell-inner plexiform layer (GCIPL) thickness, which has been increasingly recognized as an important structural marker in NAION [30], was not included in the analysis due to the limited number of patients with available GCIPL data. Future prospective studies with systematic GCIPL acquisition would help clarify its prognostic value in visual field recovery. Finally, because VF recovery was defined according to changes in MD, functional changes related to central vision may not have been fully captured. Future studies incorporating VF pattern analysis and patient-reported outcome measures may provide a more comprehensive assessment of functional recovery in NAION.

In conclusion, baseline functional status was the most important prognostic factor for VF recovery in patients with NAION. Initial VF parameters, particularly MD and VFI, were independently associated with recovery, whereas demographic factors, systemic comorbidities, and disc edema location were not. These findings suggest that, once NAION has occurred, the extent of initial VF damage may reflect the remaining functional reserve and serve as the most clinically meaningful indicator of recovery potential. Therefore, baseline VF evaluation may provide valuable information for prognostic stratification in clinical practice. Additionally, while topical agents such as brimonidine and netarsudil did not show a significant association with recovery, further research is required regarding the novel agents for the neuroprotection in patients with NAION.

## References

[1] Hayreh SS, Zimmerman B. Visual field abnormalities in nonarteritic anterior ischemic optic neuropathy: their pattern and prevalence at initial examination. Arch Ophthalmol. 2005;123(11):1554–62. doi: 10.1001/archopht.123.11.1554 16286618

[2] Hayreh SS. Ischemic optic neuropathy. Prog Retin Eye Res. 2009;28(1):34–62. doi: 10.1016/j.preteyeres.2008.11.002 19063989

[3] Kaur K, Margolin E. Nonarteritic Anterior Ischemic Optic Neuropathy. 1st ed. Treasure Island (FL): StatPearls Publishing; 2026. 32644471

[4] Martin-Gutierrez MP, Petzold A, Saihan Z. NAION or not NAION? A literature review of pathogenesis and differential diagnosis of anterior ischaemic optic neuropathies. Eye (Lond). 2024;38(3):418–25. doi: 10.1038/s41433-023-02716-4 37770527 PMC10858240

[5] Hayreh SS, Zimmerman MB. Nonarteritic anterior ischemic optic neuropathy: natural history of visual outcome. Ophthalmology. 2008;115(2):298–305.e2. doi: 10.1016/j.ophtha.2007.05.027 17698200 PMC2782939

[6] Lantos K, Dömötör ZR, Farkas N, Kiss S, Szakács Z, Garami A, et al. Efficacy of treatments in nonarteritic ischemic optic neuropathy: a systematic review and meta-analysis. Int J Environ Res Public Health. 2022;19(5):2718. doi: 10.3390/ijerph19052718 35270411 PMC8910678

[7] Miller NR, Arnold AC. Current concepts in the diagnosis, pathogenesis and management of nonarteritic anterior ischaemic optic neuropathy. Eye (Lond). 2015;29(1):65–79. doi: 10.1038/eye.2014.144 24993324 PMC4289822

[8] Biousse V, Newman NJ. Ischemic optic neuropathies. N Engl J Med. 2015;372(25):2428–36. doi: 10.1056/NEJMra1413352 26083207

[9] Hayreh SS. Pathogenesis of nonarteritic anterior ischemic optic neuropathy. Arch Ophthalmol. 2009;127(8):1082–3; author reply 1083-4. doi: 10.1001/archophthalmol.2009.199 19667362

[10] Ischemic Optic Neuropathy Decompression Trial Research Group. Ischemic optic neuropathy decompression trial: twenty-four-month update. Arch Ophthalmol. 2000;118(6):793–8. 10865316

[11] Katz DM, Trobe JD. Is there treatment for nonarteritic anterior ischemic optic neuropathy. Curr Opin Ophthalmol. 2015;26(6):458–63. doi: 10.1097/ICU.0000000000000199 26367094

[12] Sharma S, Kwan S, Fallano KA, Wang J, Miller NR, Subramanian PS. Comparison of visual outcomes of nonarteritic anterior ischemic optic neuropathy in patients with and without diabetes mellitus. Ophthalmology. 2017;124(4):450–5. doi: 10.1016/j.ophtha.2016.11.029 28017420

[13] Hayreh SS. Duke-elder lecture. Systemic arterial blood pressure and the eye. Eye (Lond). 1996;10 (Pt 1):5–28. doi: 10.1038/eye.1996.3 8763299

[14] Hayreh SS, Zimmerman MB, Podhajsky P, Alward WL. Nocturnal arterial hypotension and its role in optic nerve head and ocular ischemic disorders. Am J Ophthalmol. 1994;117(5):603–24. doi: 10.1016/s0002-9394(14)70067-4 8172267

[15] Jacobson DM, Vierkant RA, Belongia EA. Nonarteritic anterior ischemic optic neuropathy. A case-control study of potential risk factors. Arch Ophthalmol. 1997;115(11):1403–7. doi: 10.1001/archopht.1997.01100160573008 9366670

[16] Salomon O, Huna-Baron R, Kurtz S, Steinberg DM, Moisseiev J, Rosenberg N, et al. Analysis of prothrombotic and vascular risk factors in patients with nonarteritic anterior ischemic optic neuropathy. Ophthalmology. 1999;106(4):739–42. doi: 10.1016/S0161-6420(99)90159-8 10201595

[17] Deramo VA, Sergott RC, Augsburger JJ, Foroozan R, Savino PJ, Leone A. Ischemic optic neuropathy as the first manifestation of elevated cholesterol levels in young patients. Ophthalmology. 2003;110(5):1041–6; discussion 1046. doi: 10.1016/S0161-6420(03)00079-4 12750110

[18] Behbehani R, Ali A, Al-Moosa A. Risk factors and visual outcome of non-arteritic ischemic optic neuropathy (NAION): experience of a tertiary center in Kuwait. PLoS One. 2021;16(2):e0247126. doi: 10.1371/journal.pone.0247126 33600480 PMC7891726

[19] Kemchoknatee P, Arjkongharn N, Pongpirul K. Impact of lipid parameters on visual acuity change in non-arteritic anterior ischemic optic neuropathy. Clin Ophthalmol. 2024;18:3791–800. doi: 10.2147/OPTH.S500368 39712368 PMC11660657

[20] Preechawat P, Bruce BB, Newman NJ, Biousse V. Anterior ischemic optic neuropathy in patients younger than 50 years. Am J Ophthalmol. 2007;144(6):953–60. doi: 10.1016/j.ajo.2007.07.031 17854756

[21] Sun M-H, Liao YJ. Structure-function analysis of nonarteritic anterior ischemic optic neuropathy and age-related differences in outcome. J Neuroophthalmol. 2017;37(3):258–64. doi: 10.1097/WNO.0000000000000521 28538035

[22] Bindu S, Jyothi P, Prabhu P. Clinical profile and predictors of visual outcome in nonarteritic anterior ischemic optic neuropathy. Kerala J Ophthalmol. 2018;30(3):183. doi: 10.4103/kjo.kjo_47_18

[23] Hayreh SS, Zimmerman MB. Optic disc edema in non-arteritic anterior ischemic optic neuropathy. Graefes Arch Clin Exp Ophthalmol. 2007;245(8):1107–21. doi: 10.1007/s00417-006-0494-0 17219123

[24] Contreras I, Noval S, Rebolleda G, Muñoz-Negrete FJ. Follow-up of nonarteritic anterior ischemic optic neuropathy with optical coherence tomography. Ophthalmology. 2007;114(12):2338–44. doi: 10.1016/j.ophtha.2007.05.042 17719640

[25] Saylor M, McLoon LK, Harrison AR, Lee MS. Experimental and clinical evidence for brimonidine as an optic nerve and retinal neuroprotective agent: an evidence-based review. Arch Ophthalmol. 2009;127(4):402–6. doi: 10.1001/archophthalmol.2009.9 19365015

[26] Atkins EJ, Bruce BB, Newman NJ, Biousse V. Treatment of nonarteritic anterior ischemic optic neuropathy. Surv Ophthalmol. 2010;55(1):47–63. doi: 10.1016/j.survophthal.2009.06.008 20006051 PMC3721361

[27] Shaw PX, Sang A, Wang Y, Ho D, Douglas C, Dia L, et al. Topical administration of a Rock/Net inhibitor promotes retinal ganglion cell survival and axon regeneration after optic nerve injury. Exp Eye Res. 2017;158:33–42. doi: 10.1016/j.exer.2016.07.006 27443501 PMC5821900

[28] Wu Y, Zhou L-M, Lou H, Cheng J-W, Wei R-L. The association between obstructive sleep apnea and nonarteritic anterior ischemic optic neuropathy: a systematic review and meta-analysis. Curr Eye Res. 2016;41(7):987–92. doi: 10.3109/02713683.2015.1075221 26443989

[29] Budihardja BM, Anggraini E, Pratiwi RW, Nastiti AD, Nusanti S. Neuroprotective strategies for nonarteritic anterior ischemic optic neuropathy: a systematic review. Korean J Ophthalmol. 2023;37(4):328–39. doi: 10.3341/kjo.2022.0166 37563973 PMC10427903

[30] De Dompablo E, García-Montesinos J, Muñoz-Negrete FJ, Rebolleda G. Ganglion cell analysis at acute episode of nonarteritic anterior ischemic optic neuropathy to predict irreversible damage. A prospective study. Graefes Arch Clin Exp Ophthalmol. 2016;254(9):1793–800. doi: 10.1007/s00417-016-3425-8 27422787

